# Concurrent Generation of Effector and Central Memory CD8 T Cells during Vaccinia Virus Infection

**DOI:** 10.1371/journal.pone.0004089

**Published:** 2008-12-31

**Authors:** Amale Laouar, Monika Manocha, Viraga Haridas, N. Manjunath

**Affiliations:** Immune Disease Institute, Inc and Department of Pediatrics, Harvard Medical School, Boston, Massachusetts, United States of Ameirca; New York University School of Medicine, United States of America

## Abstract

It is generally thought that during the contraction phase of an acute anti-viral T cell reponse, the effector T cells that escape activation-induced cell death eventually differentiate into central memory T cells over the next several weeks. Here we report that antigen-specific CD8T cells with the phenotype and function of central memory cells develop concomitantly with effector T cells during vaccinia virus (vv) infection. As soon as 5 days after an intraperitoneal infection with vv, we could identify a subset of CD44^hi^ and CD62L^+^ vv-specific CD8 T cells in the peritoneal exudate lymphocytes. This population constituted approximately 10% of all antigen-specific T cells and like central memory T cells, they also expressed high levels of CCR7 and IL-7R but expressed little granzyme B. Importantly, upon adoptive transfer into naïve congenic hosts, CD62L^+^, but not CD62L^−^ CD8 T cells were able to expand and mediate a rapid recall response to a new vv challenge initiated 6 weeks after transfer, confirming that the CD62L^+^ vv-specific CD8 T cells are bonafide memory cells. Our results are thus consistent with the branched differentiation model, where effector and memory cells develop simultaneously. These results are likely to have implications in the context of vaccine design, particularly those based on vaccinia virus recombinants.

## Introduction

After a viral infection, naïve T cells expand enormously and differentiate into effector cells that control the infection [1], [2]. After clearance of antigen, most of the effector T cells are eliminated by activation-induced cell death (AICD). However during this process, a few cells develop into memory T cells that survive for extended periods of time and mediate a rapid and robust recall response following a subsequent infection by the same pathogen (reviewed in [3]–[5]. Based on the homing characteristics and effector functions, considerable heterogeneity seems to exist within the memory CD8 T cell population and at least two subsets have been widely described [6]–[8]. One is designated as effector-memory T cells (CD8^EM^) that do not express the lymph node homing molecules CD62L and CCR7. This cell subpopulation is highly cytolytic, express high levels of molecules required for cell killing such as granzymes, but express little IL-7R and persist after antigen clearance predominately in nonlymphoid tissues [7]. The other is called the central memory cells (CD8^CM^), which are CD62L^+^, CCR7^+^, IL-7R^+^ and are found primarily in the secondary lymphoid organs but can also reside in other tissues [7], [9], [10]. CD8^CM^ also express high levels of CD44 and IL-7R. They are also less cytolytic compared to CD8^EM^, exhibit increased survival with a capacity for antigen-independent self-renewal [6], [8], [9]. CD8^CM^ are endowed with crucial immune functions and can stimulate antigen-carrying DCs, thereby behaving as helpers for T_H_1 and cytotoxic responses [6], [8]. Furthermore, when compared to CD8^EM^ cells, CD8^CM^ cells appear to have enhanced sensitivity to antigen that leads to a rapid recall response [8]. Thus, the CD8^CM^ subpopulation might represent the most desirable memory CD8^+^ T-cell subset.

The issue of how memory CD8 T cells are generated has remained controversial [5], [11]–[17], although broadly two models are nowadays acknowledged. The linear differentiation model predicts that memory CD8^+^ T cells are direct descendants of effector cells. According to this model, after Ag-activated naive CD8^+^ T cells differentiate into cytotoxic T lymphocytes (CTL), some of these effector cells escape AICD and differentiate over several weeks to fully express the properties of central memory cells [9], [11], [15], [18]. Conversely, the branched differentiation model predicts that memory T cells are derived from a precursor that precedes CTL and differentiate through a lineage parallel to effectors [13], [14], [16], [17]. Although the current thinking based on extensive studies on LCMV infection favors a linear differentiation model, a recent study by Reiner et al using a transgenic T cell adoptive transfer model showed that memory and effector cells can arise as a result of asymmetric division of CD8 T cells, with one daughter cell becoming effector and the other daughter becoming memory cell [17]. Notably, the differential commitment of the two daughter cells is determined as early as the third division upon encounter with an antigen. This model is also consistent with our earlier study that had shown that effector and memory T cells could be derived by differential cytokine treatment of activated T cells in vitro, suggesting that effector differentiation is not a prerequisite for memory cell generation [14]. However, whether this is also true in the context of an infection in vivo is not clear. In this study, we address this issue in the context of vaccinia virus. We were able to identify antigen-specific CD8 T cells with typical features of memory T cells, concomitantly with effector T cells at early time points after infection.

## Results and Discussion

### Identification of antigen-specific CD8^+^ T cells with a memory phenotype during an acute listeria monocytogenes infection in cell transfer settings

**Figure 1:**
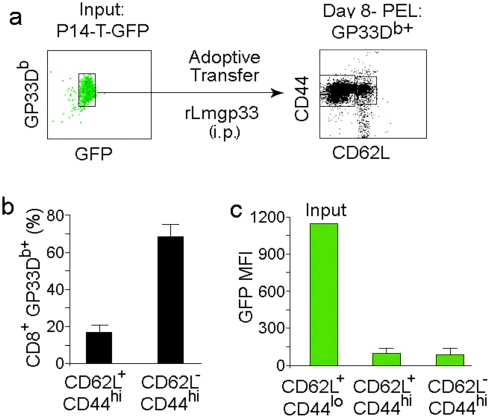
Identification of CD44^hi^ CD62L^hi^ CD8 T cell population during an acute infection with listeria monocytogenes. C57 mice were adoptively transferred with CD8 T cells isolated from uninfected naïve P14XT-GFP mice (a, input, left panel) and infected with rLmgp33. Eight days later, PELs from infected mice were examined for the presence and phenotype of transferred cells. A representative dot plot (a, right panel) and cumulative data from 6 mice (b) of CD44 and CD62L expression by CD8 and gp33D^b^ tetramer-gated cells is shown. The bar graph in (c) shows the mean fluorescence intensity (MFI) of GFP expression by the transferred input donor cells and CD62L^+^ and CD62L^−^ gp33D^b^ tetramer^+^ cells in recipient mice after infection. Data are presented as mean±s.d. from two independent experiments with 3 mice per experiment.

We initially tested if memory-like cells can be identified at early time points during Lm infection. We reasoned that if effector and memory T cells are simultaneously generated from the asymmetric division of a T cell after it responds to a microbial challenge, a subpopulation with central memory phenotype should be identified concurrently with the effector subset early after infection. To test this, we used an adoptive transfer model using CD8 T cells from P14 (specific for LCMV gp33–41) mice crossed to T-GFP mice. The advantage of this system is that it allows the detection of antigen specific cells by MHC-peptide tetramer staining and simultaneously distinguishes the naïve from activated T cells because, GFP expressed by naïve T cells is turned off once the cells start dividing after activation [14]. C57 mice were transferred with CD8 T cells isolated from naïve P14 xT-GFP mice (Fig. 1a left panel) and 3 days later, infected ip with recombinant Lm expressing gp33 epitope (rLmgp33, 10^4^ CFU). Eight days after infection, their peritoneal exudate lymphocytes (PEL) were tested for the presence and phenotype of the transferred donor cells. We chose to monitor the expression of CD62L because it is a key marker that segregates the effector and memory T cell subsets in combination with CD44, which is expressed at high levels in all effector and memory but not in naïve T cells [14], [19]. This analysis revealed the presence of two major subsets. A majority of gp33 tetramer^+^ CD8 T cells (85.3±6.7%) were CD44^+^ CD62L^−^, a profile that is reminiscent of the effector phenotype. However a significant proportion of cells (14.7±5.9%) was CD44^+^ CD62L^+^ a phenotype that generally typifies central memory cells [6], [14] (Fig. 1, a right panel and 1 b). Moreover they also had lost GFP expression (Fig. 1c), indicating that they were not input naïve cells, but were activated and had undergone cell division. Thus, our data suggest that even at the acute phase of a primary response, a population of post-mitotic CD44^hi^ CD62L^hi^ CD8^+^ T cells can be detected.

### Antigen-specific CD8^+^ T cells with a central memory phenotype can also be detected at early time points after a natural infection with vaccinia virus

**Figure 2:**
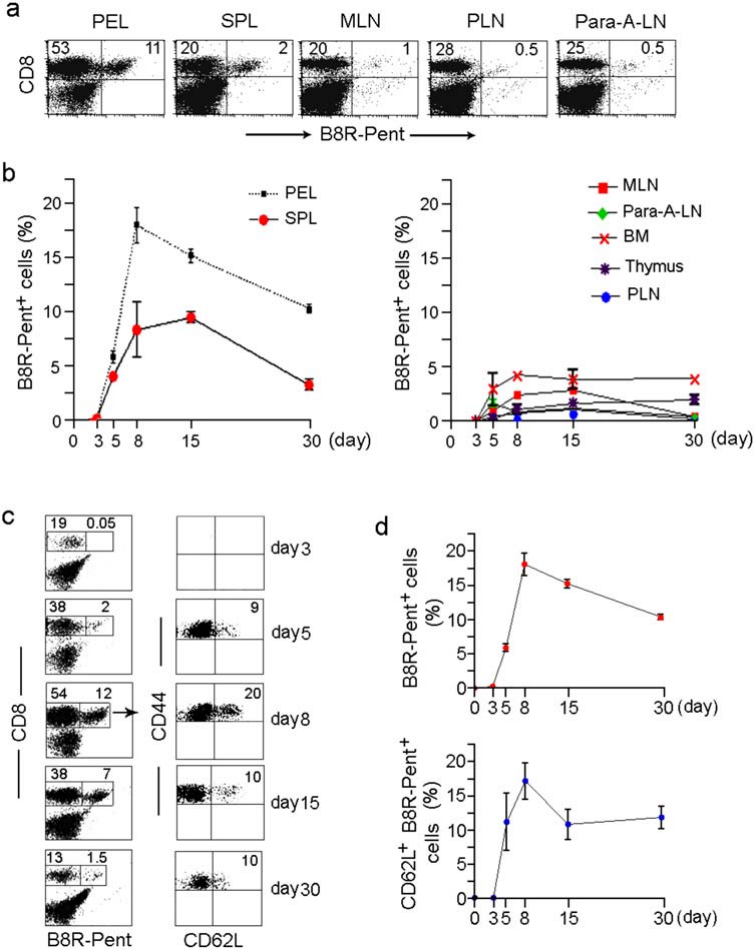
Kinetics and phenotype of antigen-specific CD8+ T response during an acute infection with vaccinia virus. C57BL/6 mice were i.p. infected with vv and at different time points after infection, the presence of B8R_20–27_ pentamer^+^ CD8^+^ T cells in different tissues was assessed by flow cytometry. Representative dot plot of results on day 8 (a) and cumulative data from 12 mice at different time points (b) on the presence of B8R_20–27_ pentamer^+^ CD8^+^ T cells are shown (SPL, spleen; BM, bone marrow; PLN, peripheral (inguinal and axillaries) lymph nodes; MLN, mesenteric lymph node; Para-A-LN, Para aortic lymph node). (c) Mice were infected with vv as in (a) and at indicated times post-infection, their PELs were tested for the presence and phenotype of B8R_20–27_ CD8^+^ T cells. Representative dot plots in the left panel show the percent of B8R_20–27_ pentamer^+^ CD8^+^ T cells and the right panel shows CD62L and CD44 expression by the B8R_20–27_ pentamer^+^ CD8^+^ gated cells (*n* = 4). (d) shows the cumulative data on B8R_20–27_ pentamer^+^ T cells as a percent of CD8 T cells in PEL (upper panel) and CD62L+ B8R_20–27_ pentamer^+^ T-cells as a percent of total B8R^+^_20–27_ T-cells (lower panel).

**Figure 3:**
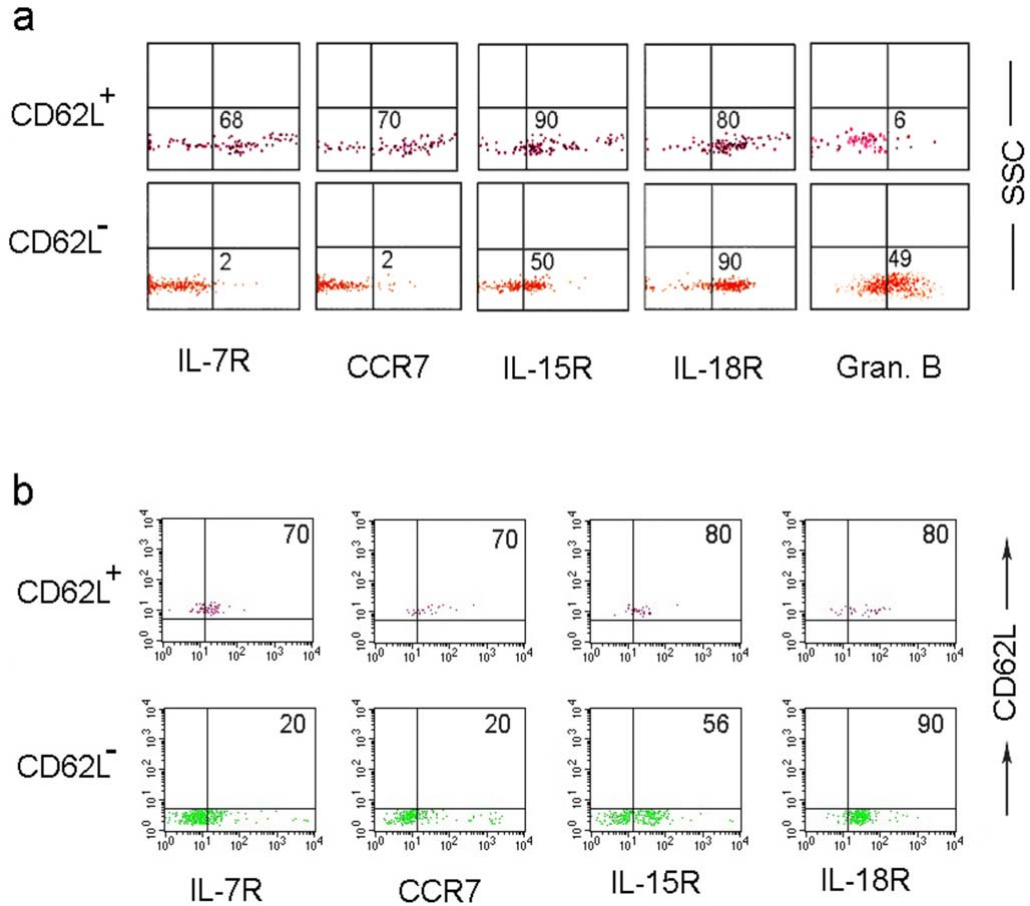
Antigen-specific CD8+ T cells with both effector and central memory phenotype can be detected during an acute infection with vaccinia virus. C57BL/6 mice were i.p. infected with vv and after 7 (a) or 5 (b) days of infection, the phenotype of CD62L^+^ and CD62L^−^ B8R_20–27_ CD8^+^ T cells in the PELs examined by flow cytometry. The indicated marker expression by CD62L^+^ and CD62L^−^ B8R+_20–27_ CD8^+^ gated T cells is shown. Results are representative of three independent experiments.

One limitation of the above experiments is that the ‘unnatural’ numbers of antigen-specific T cell precursors present in the adoptive transfer system may not accurately reflect the situation that occurs during a natural infection. For instance, because of excessive precursor numbers, the kinetics T cell differentiation may be faster than that occurring in a natural infection. Thus, to determine whether the observed CD8^+^ T cell subset with a memory phonotype (CD44^high^ CD62L^high^) also occurs under physiological conditions, we studied the antigen-specific CD8 T cell differentiation during vaccinia virus infection. First, we determined the kinetics of vaccinia-specific T cell response in different organs following an ip infection with vv using the recently described immunodominant B8R_20–27_ peptide (TSYFESV) [20] /MHC pentamers. VV-specific CD8 T cell response peaked on day 8 and the maximal response was seen in the PEL, where the B8R_20–27_ pentamer^+^ cells constituted 10–20% of CD8 T cells, followed by spleen, but few antigen-specific cells were detectable in the other lymphoid organs (Fig. 2a, b). Because the maximal response was seen in the PEL compartment, we used PEL CD8 T cells for further studies. We examined the B8R_20–27_ pentamer^+^ CD8 T cells for expression of CD62L and CD44 at different times after infection. Strikingly, ∼10–20% of B8R_20–27_ pentamer^+^ cells were CD44^hi^ and CD62L^+^ and these cells could be detected as soon as any pentamer positive cells could be detected, starting on day 5 after infection (Fig. 2c). Moreover, they were continually present throughout the 30-day observation period (Fig. 2c, d). CD62L^+^ cells peaked on day 8 (constituting ∼20%) and remained at approximately 10% of B8R_20–27_ pentamer^+^ cells throughout the study period (Fig. 2c, d). Thus, antigen-specific T cells with CD62L^+^ CD44^hi^ phenotype can also be detected at early periods during a normal immune response to a viral challenge. To our knowledge, this is the first report of detection of CD62L^+^ pentamer^+^ antigen specific CD8 T cells during an acute infection. We further characterized this subset using markers that differentiate naïve, effector and memory T cells in a four-color flow cytometric analysis. On day 7 post-infection (at the peak of response), the CD62L negative and CD62L positive B8R_20–27_ pentamer+ CD8 gated cells were examined for the expression of CCR7, IL-7R, IL-15R, IL-18R (by external staining) and granzyme B (by internal staining). As Fig. 3a shows, a majority of CD62L^−^ B8R_20–27_ pentamer^+^ cells did not express CCR7 or IL-7R, but expressed IL-15R, IL-18R and granzyme B, typical of effector cells [8], [21]. In contrast, a majority of CD62L^+^ B8R_20–27_ pentamer^+^ cells expressed CCR7 and IL-7R but little granzyme B. However they also expressed IL-15R and IL-18R. We also tested the phenotypic profile of CD62L^+^ and CD62L^−^ B8R_20–27_ pentamer+ cells on day 5 post-infection, which is the earliest time at which any B8R_20–27_ pentamer^+^ CD8 T cells could be discerned (Fig.2c). Even here, the CD62L^+^ antigen-specific cells expressed CCR7 and IL-7R while the majority of CD62L^−^ cells were CCR7-, IL7R- (Fig. 3b). Thus, antigen-specific CD8 T cells exhibiting a central memory phenotype can be detected as soon as 5 days after a vaccinia infection. Although naïve T cells also express CCR7 and IL-7R and do not express granzyme B, the CD62L^+^ B8R_20–27_ pentamer^+^ cells are unlikely to be naïve cells because they expressed high levels of CD44, IL-15R and IL-18R (Fig. 3a, b) and NKG2A (data not shown). Moreover, because naïve T cells specific for a given TCR specificity are thought be extremely few (estimated as 10–100 cells/animal in mice) [2], [22], no study has reported the presence of naïve antigen-specific T cells during an infection. Further, naïve T cells circulate from blood to lymphoid organs and are thought not to traffic to tissue sites of infection such as PEL [23]. Additionally, it is highly unlikely that naïve cells are being recruited into the peritoneal cavity even after the clearance of the antigen (typically vaccinia infection is resolved completely in 10–14 days) [24]. Taken together, our results strongly suggest that the CD62L^+^ B8R2_20–27_ pentamer^+^ cells are not naïve, but have already been activated with antigen and express the phenotypic features of central memory T cells and that this cell population can be found concomitantly with effector T cells during a primary immune response.

### CD62L^+^ B8R_20–27_ pentamer^+^ cells mount long-term memory response to rechallenge with vv

**Figure 4:**
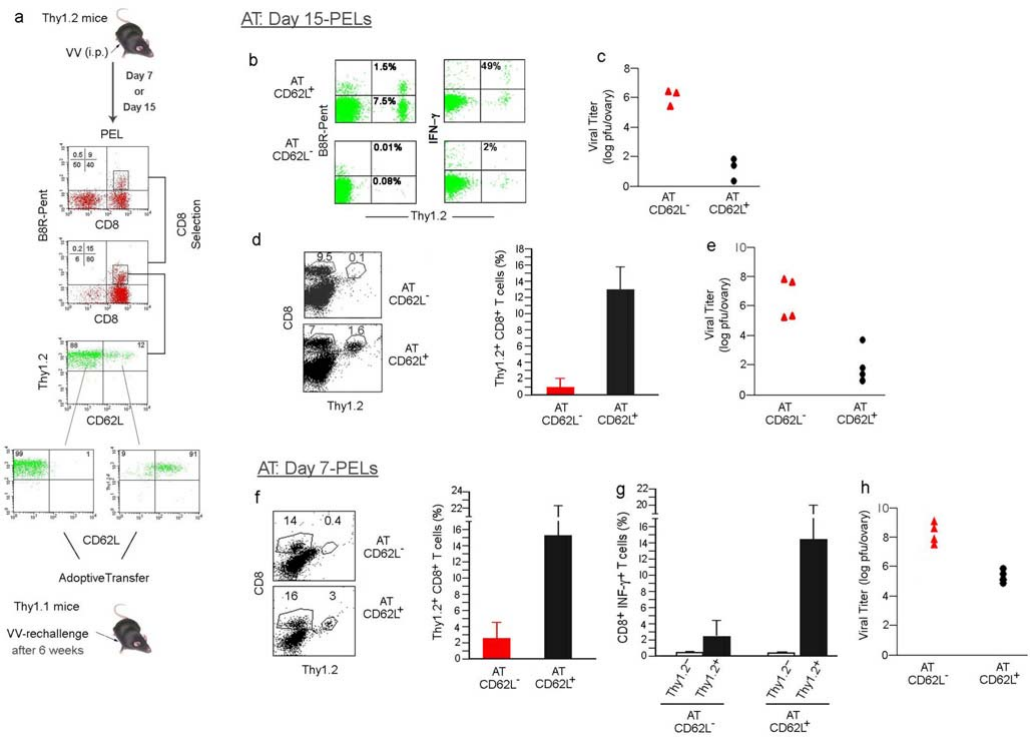
CD62L^+^ CD8 T cells isolated from vv-infected mice survive for long periods of time and mount a rapid recall response. (a) Experimental approach for adoptive transfer experiments. PELs were harvested from day 7 or 15 vv-infected C57 (H-2D^b^, Thy1.2^+^) mice, CD8^+^ T cells were negatively selected and the presence of CD62L^+^ and CD62L^−^ B8R^+^_20–27_ CD8^+^ cells confirmed. The CD62L^−^ and CD62L^+^ CD8^+^ T-cell subsets were further isolated (purity of isolated cells is shown in the bottom dot plots) and injected into naïve sex-matched congenic recipient C57 (H-2D^b^, Thy1.1^+^) mice and the mice rested for different times before challenging with vv. (b) After 8 days of the transfer, the recipient mice were infected with vv and 3 days later, their PELs were tested for the presence of donor-derived Thy1.2^+^ B8R^+^_20–27_ CD8^+^ T cells (left panel) or tested for intracellular IFN-γ production after ex vivo stimulation with B8R peptide-pulsed DC (right panel). One representative result from three mice each, transferred with CD62L^−^ or CD62L^+^ CD8 T cells are shown. The numbers within the dot plots in the left panel represent percentage of total cells in the PEL and in the right panel, they represent percentage of cytokine+ cells among all donor cells (c) Viral titer from the ovaries of the same recipient mice in (b) is shown (n = 3). (d) Six weeks after of the transfer of cells obtained from day 15 vv-infected mice, the recipient mice were challenged with vv and after 3 days, the presence of Thy1.2^+^ donor-derived CD8 T cells tested in the CD62L^−^ and CD62L^+^ CD8 T cell transferred mice. Representative dot plot (left panel) and cumulative data from four mice on the presence of donor derived Thy1.2^+^ CD8 T cells are shown. The numbers within the dot plots in the left panel represent percentage of total cells in the PEL. (e) Viral titers from the ovaries of the same recipient mice in (d) is shown (*n* = 4). (f) After 6 weeks of the transfer of cells obtained from day 7 vv-infected mice, the recipient mice were challenged with vv and after 3 days, the presence of Thy1.2^+^ donor-derived CD8 T cells in the PELs were tested in the CD62L^−^ and CD62L^+^ CD8 T cell transferred mice. Representative dot plot (left panel) and cumulative data from four mice on the presence of donor derived Thy1.2^+^ CD8 T cells are shown. The numbers within the dot plots in the left panel represent percentage of total cells in the PEL. (g) IFNγ production by CD8 T cells in mice described in (f) is shown. (h) Viral titers from the ovaries of the same recipient mice in (f) is shown (*n* = 4).

A hallmark of memory CD8 T cells is long-term survival and ability to mediate a rapid recall response following rechallenge with the pathogen. Because the CD62L^+^ B8R_20–27_ pentamer^+^ cells exhibited the phenotypic characteristics of central memory cells, we tested if they are also capable of memory functions. Since the presence of neutralizing antibodies may complicate the analysis of CD8 T cell memory function during a second infection in the same mouse [24], we performed adoptive transfer experiments for these studies using congeneic mice (Fig. 4a). We first tested the potential for in vivo survival and function of CD62L^+^ and CD62L^−^ CD8 T cell populations after transfer to naïve uninfected recipient mice. C57 (Thy1.2) mice were infected with vv and 15 days later, their PEL CD8 T cells were negatively selected using the R&D kit. The CD62L^−^ and CD62L^+^ B8R_20–27_ pentamer positive cells constituted ∼10 and ∼0.9% respectively of PEL CD8 T cells. The CD8 T cells were further immunomagnetically sorted and equivalent numbers (10^6^ donor cells/mouse) of CD62L^−^ and CD62L^+^ CD8 T cells (C57, Thy1.2) were i.v. injected into naive congeneic mice (C57, Thy1.1^+^). Because TCR cross linking might restimulate the cells, we did not use pentamer staining for sorting the cells. Since the CD62L^+^ B8R_20–27_ pentamer^+^ cells constituted 10% of total B8R_20–27_ pentamer^+^ CD8 T cells in the PEL and we transferred equal numbers of CD62L^−^ and CD62L^+^ CD8 T cells, we actually transferred ∼10 times more CD62L^−^ than CD62L^+^ B8R_20–27_ pentamer^+^ cells/recipient mouse (equivalent to ∼10^5^ CD62L^−^ and 10^4^ CD62L^+^ pentamer^+^ cells). To test the survival and proliferative capacity of transferred donor cells, the recipient mice were challenged 8 days after transfer with vv i.p. and their PELs were examined for Thy1.2^+^ donor-derived pentamer+ cells 3 days after viral challenge. Despite the fact that ∼10 times more CD62L^−^ than CD62L^+^ cells had been transferred, pentamer^+^ CD8 T cells of donor origin (Thy1.2) could only be detected in the CD62L^+^ cell transferred mice (Fig. 4b, left panel), suggesting that the CD62L^+^, but not CD62L^−^ transferred cells could survive in a naïve host. The CD62L- cells isolated during the acute phase of infection failed to survive in antigen-naïve hosts after transfer probably because they represent fully differentiated effector cells that are destined to die upon sudden withdrawal from antigen and/or the cytokine milieu associated with infection (a situation different from the endogenous CD62L- cells seen at late periods after infection shown in Fig. 2c, which probably represent “effector memory” cells). In contrast, the CD62L+ cells were capable of survival probably because they represent central memory type of cells that are capable of survival in the absence of antigen. Moreover, a substantial portion of CD62L^+^ donor cells were also capable of interferon-γ production in response to ex-vivo stimulation with B8R_20–27_ peptide (Fig. 3b, right panel). In addition, the viral burden in the CD62L^+^ CD8 T cell transferred mice was substantially lower compared to CD62L^−^ CD8 T cell transferred mice (Fig. 4c). The protection observed in the CD62L+ cell transferred mice is not due to an endogenous host response elicited by coincidental transfer of vv at the time of adoptive transfer because: (1) we were unable to isolate the virus from sorted cells used for transfer (data not shown) and (2) since the CD62L+ or CD62L- cells isolated from the same infected mice were used for transfer to separate recipient mice, if virus was transferred along with donor cells, both groups of recipient mice should have developed a protective immune response leading to the presence of a substantial number of endogenous (Thy1.2-) pentamer+ CD8 T cells and lack of virus upon rechallenge. However, while there was no significant expansion of endogenous pentamer+ cells in both groups of mice (Fig. 4b), there was high level virus replication in CD62L- T cell transferred, but not in CD62L+ cell transferred recipients. Taken together, these results show that while the antigen-specific effector cells generated early after vv infection are destined to die in an antigen free environment, the CD62L^+^ memory-like cells can survive and mediate effector function following a reinfection.

Although the above results suggest that the CD62L^+^ cells generated early after vaccinia virus infection survive in vivo for at least 8 days and mount an effector response to rechallenge, true memory cells should be capable of long-term survival. To test this, we repeated the adoptive transfer experiments and this time rested the recipient mice for 6 weeks before challenging with vv infection. Even under these conditions, it is only the CD62L^+^ transferred cells that showed significant expansion (Fig. 4d). The donor derived CD62L^+^ cells detected appear to be vaccinia-specific because when we transferred similar numbers of CD62L+ CD8 T cells from naïve (vv uninfected) mice, we could hardly detect any donor cells in the PEL of vv infected recipients (data not shown). Moreover, the recipient mice that received the CD62L^+^ cells (from vv-infected donors) showed significantly reduced viral burden compared to CD62L^−^ cell transferred mice (Fig. 4e). Thus, the CD62L^+^ CD8 T cells generated after 15 days of infection appear to be memory T cells.

It has recently been suggested that memory precursors can be identified within the effector cell population at early time points after infection and that the conversion of effector to central memory CD8 T cells occurs gradually starting from the first week of stimulation in vivo [9], [25].Thus a possibility remained that by day 15 after vv infection, memory cells could have arisen from differentiated effector T cells. To test this possibility, we repeated the adoptive transfer experiments using effector and memory phenotype cells isolated from mice 7 days after infection (the limited number of antigen-specific T cells produced precluded us from using the earliest time point of post-infection day 5). CD62L^+^ and CD62L^−^ CD8 T cells from the PELs of 7 day vv-infected Thy1.2 mice was isolated by FCAS-based cell sorting and transferred to naïve Thy1.1 mice. The recipient mice were rested for 6 weeks, infected with vv and 3 days later their PEL examined for the presence of donor-derived Thy1.2^+^ CD8 T cells. Even here, a significant number of donor-derived CD8 T cells and interferon γ production could be detected in the CD62L^+^ cell transferred mice but not in the CD62L^−^ cell transferred mice (Fig. 4f, g). Moreover, viral titers were also lower in the CD62L^+^ cell transferred mice compared to CD62L^−^ cell transferred mice (Fig. 4h). Thus, as early as 7 days after an acute vaccinia infection, central memory-like CD8 T cells, with capacity to survive in the absence of antigen for long periods of time and to expand and protect against a reinfection can be detected.

Taken together our results suggest that central memory CD8 T cells are generated simultaneously with effector T cells during vaccinia infection. These results are in agreement with the studies from the Reiner's group which found that memory CD8 T cells arise by asymmetric division of antigen-stimulated cells, implying that both cell types can arise from a common progenitor very early in the response to infection [17]. However it should be cautioned that from our studies, we can not definitively conclude that the same antigen-specific CD8 T cell precursor is giving rise to both effector and memory cells.

Unlike our results with vaccinia infection, cells with the phenotypic and functional characteristic of central memory cells are not seen in the acute phase of LCMV infection and based on those results, it has been concluded that memory cells are descendants of effector cells and develop gradually after the clearance of antigen [25]. Why is the situation is different in vaccina infection is not clear. However, numerous variables including antigen density and persistence, costimulation, cytokines and CD4 help are known to determine the quantity, quality and location of memory cells [26]. Although both vv and LCMV are acute infections, the antigen load and persistence is more in LCMV than vv. Moreover, the induction of costimulatory molecules as well as the response to blockade of costimulatory interaction also differs between LCMV and vv [27]. Furthermore, we have previously shown that cytokines IL-2 and IL-15 can differentially regulate the fate of antigen activated CD8 T cells into becoming either effector or memory cells [14]. Thus, it is possible that differences in the antigen density/costiumlatory signals or cytokine response between LCMV and vaccinia infection could account for the differences between our results and those reported with LCMV in the literature. A study by Bachman et al also reported that the balance between effector and memory T cells is governed by the degree of antigen load and time [28]. However, using gp33-specifc TCR Tg CD8 T cells as surrogate antigen-specific cells, they found that although CD62L+ memory cells proliferate in vivo upon infection with both LCMV and vaccinia, they clear LCMV but not vaccinia efficiently. This is not consistent with our results because we found both proliferation and protection to be mediated by central memory rather than effector cells following vaccinia rechallenge. Differences in the experimental systems might account for these differences. For e.g. Bachman et al used LCMV gp33-specifc TCR Tg memory CD8 T cells generated during an LCMV infection as surrogate vaccinia-specific cells to detect responses against a recombinant vaccinia expressing gp33 (and thus are looking at one surrogate epitope-responding T cells), we used memory CD8T cells during a native vaccinia infection (containing all native vaccinia-responding cells) to measure protection following rechallenge. Similarly, Richards et al also used a influenza NP68 epitope-specific T cells as surrogate vaccinia specific T cells to measure responses to a recombinant vv expressing NP68 epitope and found that proteolytic cleavage of CD62L is required for efficient clearance of vaccinia infection [29]. This is however, not inconsistent with our results since the wild type vaccinia-specific T cells (in this study) are not defective for CD62L cleavage.

Finally, it is also noteworthy that most studies on LCMV have focused on events in the spleen after an iv infection, while we characterized the cell populations after vaccinia infection in the peritoneal cavity after an ip infection. Thus, another possibility is that the activated CD8 T cell fate may also vary with different routes of infection and/or at different tissue sites of immune response. Further studies are needed to test these hypotheses, which may have implications particularly in improving the design of vaccinia-based vaccines.

## Materials and Methods

### Mice

C57BL/6 (C57; H-2D^b^, Thy1.2^+^), C57BL/6 (C57; H-2D^b^, Thy1.1^+^) were purchased from Jackson Laboratory. P14 X T-GFP mice have been described [14]. All mice were maintained in specific pathogen free conditions and used when they were 6–10 weeks of age. All animal experiments had been approved by the Institutional Review Board of the Immune Disease Institute (Formerly CBR Institute for Biomedical Research).

### MHC-peptide tetramers, Pentamers and antibodies

MHC H-2D^b^ gp33 tetramers were obtained from Biosource International and MHC H-2D^b^ Pro5 ^Tm^ Pentamer (B8R peptide: TSYFESV) was custom synthesized at ProImmune. Antibodies to mouse CD8, Thy1.1, Thy1.2, CD44, CD62L, and INF-γ were purchased from BD PharMingen. Antibodies to CCR7 and IL7R were from eBioscience. Antibodies to IL-15R and IL-18R were from R&D Systems. Immunostaining and flow cytometric analysis of cell-surface phenotypes and intracellular cytokines were done as described earlier [30].

### Adoptive transfer and listeria infection

These were done as described earlier [30]. Breifly, naive CD8^+^ T cells were purified from the splenocytes of P14xT-GFP mice [14] by negative selection using the murine CD8 subset isolation kit (R&D Systems) and wild type C57 mice were injected i.v. with 8×10^6^ purified CD8 T cells. On day 3 post-transfer, recipient mice were injected i.p. with 10^4^ CFU of a recombinant strain of *Listeria* monocytogenes that expresses the LCMV gp_33–41_ epitope (rLmgp33) and, at day 8 postinfection, their peritoneal exudates lymphocytes (PELs) were harvested.

#### Viral infection

Mice were infected ip with 10^6^ PFU/mouse in 200 µl PBS of vv (WR strain, ATCC) and at different times after infection their PELs, spleens, and other lymphoid organs were harvested for experiments.

### Cell sorting and adoptive transfer studies

Cells harvested from the PELs of infected C57 mice (H-2D^b^, Thy1.2^+^), were first enriched for CD8+ T cells using the murine CD8 subset isolation kit (R&D Systems). Negatively selected CD8 T cells were then stained with CD62L-PE, followed by incubation with PE-coated Miltenyi beads. Positively and negatively selected cells were subsequently isolated using a Miltenyi miniMACS system. In some experiments (to test memory function after 7 days of infection), the CD62L^+^ and CD62L^−^ CD8 T cells were FACS sorted using FACSAria cell sorter (Becton Dickinson, San Jose, CA). Age and sex-matched recipient C57 (H-2Db, Thy1.1+) mice were i.v. injected with (1×10^6^ cells/mouse) purified CD62L^+^ or CD62L^−^ cells. At day 8 or 6 weeks post-transfer, recipient mice were i.p. challenged with vaccinia virus (10^6^ PFU/mouse in 200 µl PBS) and 3 days later, their peritoneal exudates lymphocytes (PELs) and ovaries were harvested. When examining the recipient mice PELs for the presence of donor derived T cells, the cells were also stained with Thy1.2 Abs to distinguish them from the Thy1.1 host CD8 T cells. For testing IFN-γ response, syngeneic bone marrow-derived DC were pulsed with B8R_20-27_ peptide (TSYFESV; 5 µg/ml; synthesized at ProImmune), washed and used to stimulate PELs of secondary recipient mice. Non peptide-pulsed BMDC served as controls. After 18 hours of the co-cultures, the intracellular IFN-γ production was measured as described previously [30].

### Virus titration

Serial dilutions of homogenates of ovaries harvested from recipient mice were inoculated on CV-1 cells and after 2 days, stained with neutral red/formalin and the plaques were counted manually.

### Statistical analysis

Differences in values between experimental groups were examined for significance with Graph Pad Prism software using Student *t* test. We considered probability (*P*) values <0.05 as significant. Values are presented as means±SEMs.
